# Extracellular Vesicles Derived from Human Liver Stem Cells Attenuate Chronic Kidney Disease Development in an In Vivo Experimental Model of Renal Ischemia and Reperfusion Injury

**DOI:** 10.3390/ijms23031485

**Published:** 2022-01-27

**Authors:** Stefania Bruno, Giulia Chiabotto, Massimo Cedrino, Elena Ceccotti, Chiara Pasquino, Samuela De Rosa, Cristina Grange, Stefania Tritta, Giovanni Camussi

**Affiliations:** 1 Department of Medical Sciences, University of Torino, 10126 Torino, Italy; giulia.chiabotto@unito.it (G.C.); elena.ceccotti@unito.it (E.C.); chiara.pasquino@unito.it (C.P.); samuela.derosa@edu.unito.it (S.D.R.); cristina.grange@unito.it (C.G.); giovanni.camussi@unito.it (G.C.); 2 Molecular Biotechnology Center, University of Torino, 10126 Torino, Italy; massimo.cedrino@unito.it (M.C.); stefania.tritta@unito.it (S.T.); 3 Unicyte S.r.l., 10126 Torino, Italy

**Keywords:** acute kidney injury, chronic kidney disease, renal fibrosis, epithelial-to-mesenchymal transition

## Abstract

The potential therapeutic effect of extracellular vesicles (EVs) that are derived from human liver stem cells (HLSCs) has been tested in an in vivo model of renal ischemia and reperfusion injury (IRI), that induce the development of chronic kidney disease (CKD). EVs were administered intravenously immediately after the IRI and three days later, then their effect was tested at different time points to evaluate how EV-treatment might interfere with fibrosis development. In IRI-mice that were sacrificed two months after the injury, EV- treatment decreased the development of interstitial fibrosis at the histological and molecular levels. Furthermore, the expression levels of pro-inflammatory genes and of epithelial–mesenchymal transition (EMT) genes were significantly reverted by EV-treatment. In IRI-mice that were sacrificed at early time points (two and three days after the injury), functional and histological analyses showed that EV-treatment induced an amelioration of the acute kidney injury (AKI) that was induced by IRI. Interestingly, at the molecular level, a reduction of pro-fibrotic and EMT-genes in sacrificed IRI-mice was observed at days two and three after the injury. These data indicate that in renal IRI, treatment with HLSC-derived EVs improves AKI and interferes with the development of subsequent CKD by modulating the genes that are involved in fibrosis and EMT.

## 1. Introduction

Renal ischemia and reperfusion injury (IRI) results from a metabolic imbalance between the lack of oxygen and nutrients and the accumulation of waste products in the kidney. IRI is considered the most common cause of acute kidney injury (AKI), and is often related to sepsis and surgical procedures that are associated with kidney transplant [1]. Oxidative stress and inflammation play critical roles in AKI onset, leading to apoptosis and necrosis of tubular epithelial cells (TECs), with a consequent rapid loss of renal function and morphology [2]. Additionally, the maladaptive repair after AKI increases the risk of developing fibrosis and causes a predisposition to the progression to chronic kidney disease (CKD) [3,4]. To better investigate the factors linking AKI to CKD, murine models of IRI that mimic the pathogenesis of ischemic AKI with the subsequent development of CKD, are preferable.

In the context of renal fibrosis, the release of pro-fibrotic stimuli favors the establishment of a fibrogenic niche in the interstitial space and drives the epithelial–mesenchymal transition (EMT) [5]. This process triggers biochemical and phenotypical changes in fibroblasts and TECs, which converts into myofibroblasts and increases the production of transforming growth factor-beta (TGF-beta) and extracellular matrix components, thus perpetuating the tubulointerstitial fibrosis [5,6,7]. Therefore, new therapeutic approaches to reduce or even reverse the degeneration of kidney function and the progression of fibrosis are urgently needed.

Mesenchymal stromal cell (MSC)-based therapies have shown promising results in the field of the regenerative medicine [8,9]. MSCs coordinate tissue repair through the secretion of extracellular vesicles (EVs), which are involved in cell–cell communication by shuttling a variety of bioactive molecules (proteins, lipids, and nucleic acids) [10].

In recent years, EVs that were derived from MSCs (MSC-EVs) have shown great efficacy in promoting renal regeneration after damage [11]. In experimental models of AKI, the administration of MSC-EVs mimics the effects of the cell of origin, inducing amelioration of renal function and morphology [12,13,14,15,16,17,18,19,20,21,22,23,24,25,26]. In CKD mouse models, treatment with MSC-EVs improves histopathological changes and reduces fibrosis and inflammation [18,27,28,29,30,31,32,33].

Human liver stem cells (HLSCs), a liver-derived MSC-like population [34,35], have already proven effective in prompting kidney regeneration after damage. In AKI, HLSCs, contribute to the functional and histological recovery of renal damage [20]. Interestingly, HLSC-derived conditioned medium (CM) mimicked the effect of the cells by inducing improvement of renal function and morphology. However, EV depletion from the CM prevented the pro-regenerative effects that were mediated by HLSCs, thus indicating that EVs that were derived from HLSCs (HLSC-EVs) play an important role in favoring renal tissue regeneration after AKI [20]. In addition, HLSC-EVs can also improve renal function and morphology in different models of CKD, hindering the progression of renal fibrosis that develops in murine models of aristolochic acid nephropathy [32] and diabetic nephropathy [28]. The treatment of CKD mice with multiple injections of HLSC-EVs significantly reduces the expression levels of pro-fibrotic genes, such as alpha-smooth muscle actin (*alpha-SMA*), collagen I, and *TGF-beta* [28,32].

Here, we investigated the effect of HLSC-EVs in an in vivo IRI model of AKI that allows for the subsequent development of CKD.

## 2. Results

### 2.1. HLSC-EVs Improve Kidney Function and Morphology of IRI-CKD Mice

**Figure 1:**
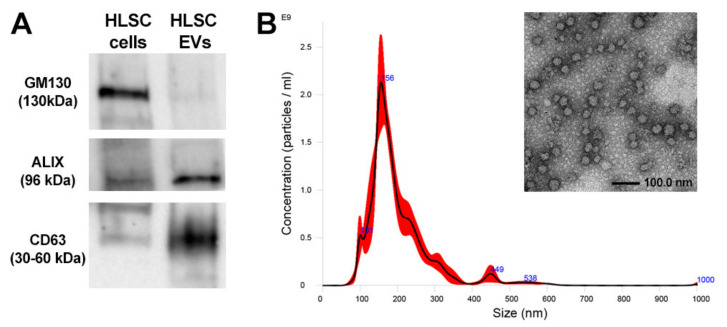
The characterization of HLSC-EVs. (**A**) Representative the Western blot analysis of exosomal markers (ALIX and CD63) in EVs. As negative control, the cis-Golgi marker GM130 was detected only in the cells, but not in the EVs. (**B**) Representative graphs of nanoparticle tracking analysis showing the size distribution of HLSC-EVs, and representative micrograph of transmission electron microscopy of the EVs that were negatively stained with NanoVan (scale bar, 100 nm; magnification, 50,000×).

To characterize HLSC-EVs, the expression of the commonly used exosomal markers ALIX and CD63 was detected by Western blot (Figure 1A). Cytofluorimetric analysis confirmed the presence of tetraspanins (CD63, CD81, and CD9). Furthermore, HLSC-EVs expressed mesenchymal markers CD29, CD44, and CD105, but did not express endothelial (CD31), hematopoietic (CD45, CD3, CD8, etc.), or epithelial (CD24 and CD326) markers [data not shown], as previously reported [36,37,38]. Nanoparticle tracking analysis on EVs revealed the typical size distribution of exosomes and shedding vesicles and transmission electron microscopy analysis confirmed that the EVs had a homogeneous pattern of nano-sized membrane vesicles (Figure 1B). 

**Figure 2:**
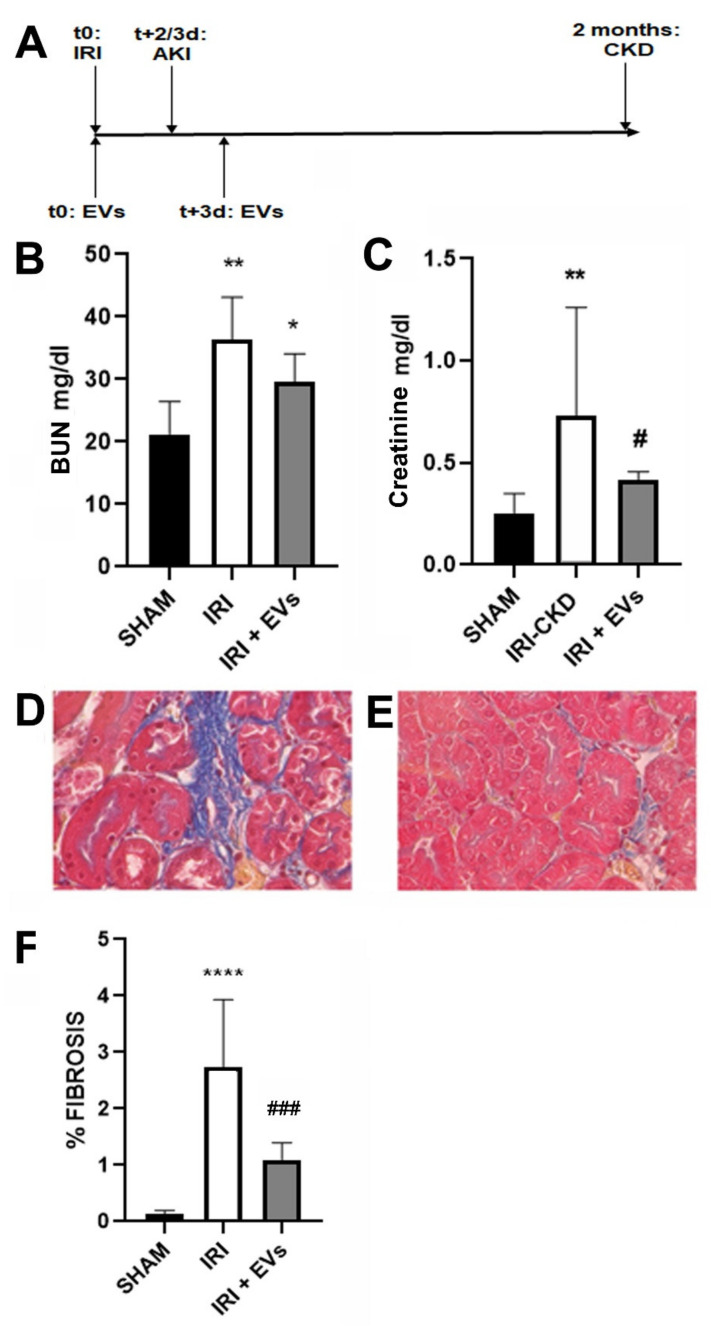
Effects of HLSC-EVs on kidney function and morphology in IRI-mice that were sacrificed two months after the surgery. (**A**) Schematic representation of the experimental design to test HLSC-EVs in an IRI murine model of CKD development, showing the time of surgery, of HLSC-EV administration, and of sacrifice. (**B**,**C**) Blood urea nitrogen (BUN) (**B**) and creatinine (**C**) plasma levels that are expressed as mg/dL were measured as biomarkers of renal function of SHAM control mice (SHAM, *n* = 5) and of IRI-mice i.v.-injected with HLSC-EVs (IRI + EVs, *n* = 8) or with the vehicle alone (IRI, *n* = 8) and sacrificed two months after the surgery. The data that are shown for BUN and creatinine analysis are represented as the mean ± SD. Statistical analysis was performed using analysis of variance (ANOVA) with Tukey’s multicomparison test: * *p* < 0.05 IRI + EVs vs SHAM and ** *p* < 0.002 IRI-mice vs SHAM for BUN analysis; ** *p* < 0.01 IRI vs SHAM and # *p* < 0.05 IRI + EVs vs IRI-mice for creatinine analysis. (**D**,**E**) Representative light microscopy micrographs of renal histology of IRI-mice IV-injected with the vehicle alone (**D**) or with HLSC-EVs (**E**) two months after the surgery (original magnification: 400×). (**F**) Histological quantification of fibrosis in SHAM, IRI + EVs, and IRI-mice that were sacrificed two months after the surgery, expressed as percentage of fibrosis within the total area and evaluated by multiphase image analysis of 10 fields per section (original magnification: 400×). The data are represented as the mean ± SD. Statistical analysis was performed using ANOVA with Tukey’s multicomparison test: **** *p* < 0.0001 IRI vs SHAM mice and ### *p* < 0.001 IRI + EVs mice vs IRI-mice.

To evaluate the potential ability of HLSC-EVs to interfere with the development of renal fibrosis, we induced 30 min-IRI with contralateral nephrectomy in male BALB-c mice. EVs (1 × 10^9^) were injected intravenously (i.v.) immediately after the surgery and again three days later (Figure 2A). IRI-mice that were sacrificed two months after the injury developed chronic kidney disease that was characterized by worsening renal function and morphology (Figure 2). 

The EV injection induced a reduction of the plasma markers of renal dysfunction (Figure 2B,C), which reached the statistical significance for plasma creatinine level (Figure 2C). At the histological level, Masson’s trichrome-staining showed interstitial fibrosis development in IRI-mice that were sacrificed two months after the surgery that was significantly reduced by EV-treatment (Figure 2D–F). 

**Figure 3:**
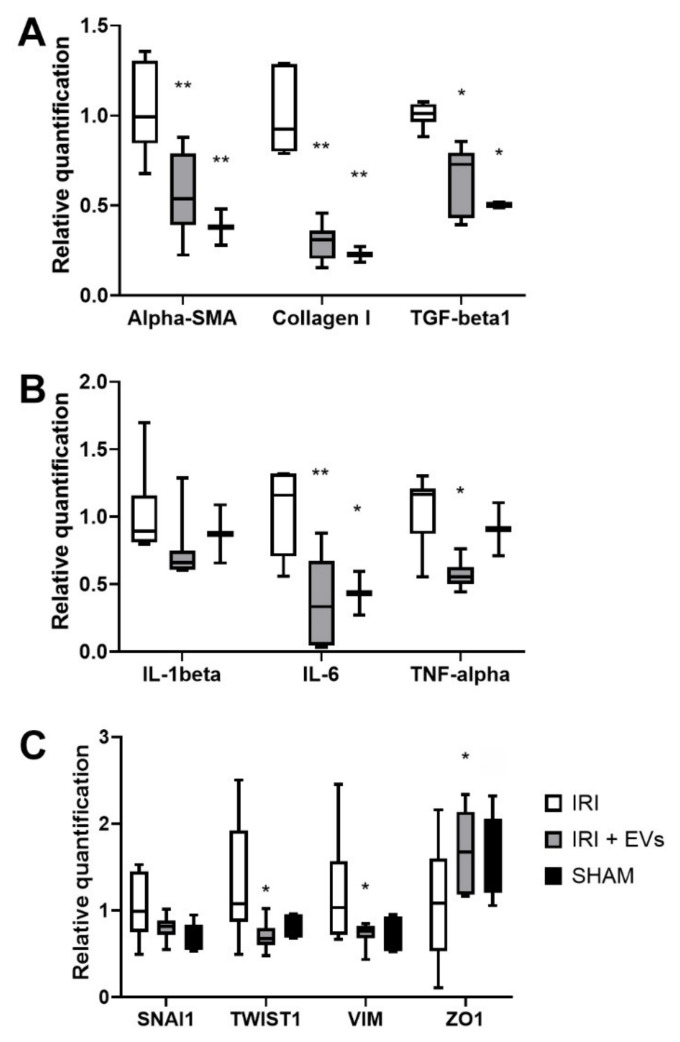
The effect of HLSC-EVs on fibrosis development in a renal IRI murine model. (**A**–**C**) Real-time PCR analysis of the expression of pro-fibrotic (*alpha-SMA, collagen I,* and *TGF-beta*) (**A**), of pro-inflammatory (*IL-1beta, IL-6*, and *TNF-alpha*) (**B**), and of EMT genes (*SNAI1, TWIST1, VIM*, and *ZO1*) (**C**) in kidneys of the SHAM control mice (SHAM, *n* = 5) and of IRI-mice that were IV-injected with HLSC-EVs (IRI+EVs, *n* = 8) or with the vehicle alone (IRI, *n* = 8). The mean ± SD was calculated using the ΔΔCt method, by comparing the gene expression levels of each group with the ones of the IRI group. Normalization was performed using *GAPDH* as the housekeeping gene. Statistical analysis was performed using ANOVA with Tukey’s multicomparison test: * *p* < 0.05, ** *p* < 0.001 SHAM and IRI + EVs vs IRI-mice.

RNA was isolated from kidney tissues of IRI-mice that were treated or not treated with EVs. Molecular analyses indicated that the IRI-mice had significantly up-regulated levels of *alpha-SMA*, *collagen I*, and *TGF-beta* (Figure 3A). Furthermore, we also observed an up-regulation of the pro-inflammatory genes interleukin-6 (*IL-6*) and tumor necrosis factor-alpha (*TNF-alpha*) (Figure 3B). In contrast, IRI-mice that were injected with EVs had a significant reduction in the expression levels of all the tested pro-fibrotic and pro-inflammatory genes (Figure 3A,B). 

**Figure 4:**
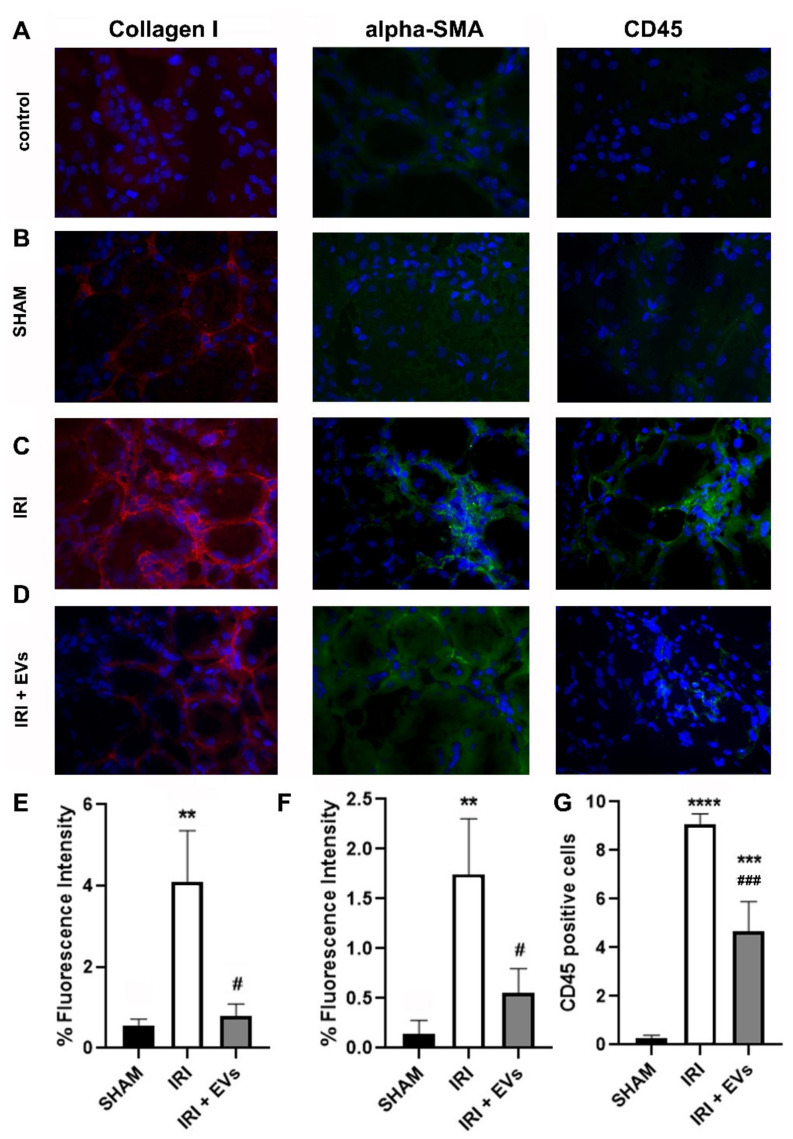
The effect of HLSC-EVs on fibrosis and inflammation in a renal IRI murine model. (**A**–**D**) Kidney cryo-sections from control (SHAM) mice (**B**), IRI-mice treated with the vehicle (**C**), or with EVs (**D**) were stained for collagen I, alpha-SMA, and CD45. Sections that were stained with secondary antibodies only served as control (**A**). (**E**–**G**) Histograms depicting the fluorescence intensity of collagen I (**E**) and alpha-SMA (**F**), and the quantification of CD45-positive cells (**G**) in renal cryo-sections from the different experimental groups. The data represent the mean ± SD of the fluorescence intensity or cells positive per high power field (HPF) that was measured from 10 images that were taken at random from four mice/groups (original magnification: 400×). Statistical analysis was performed using ANOVA with Tukey’s multicomparison test. ** *p* < 0.005 IRI-mice vs SHAM and # *p* < 0.005 IRI + EVs mice vs IRI-mice for % of fluorescence intensity for Collagen I-staining; ** *p* < 0.005 IRI-mice vs SHAM and # *p* < 0.02 IRI + EVs mice vs IRI-mice for % of fluorescence intensity for alpha-SMA-staining; **** *p* < 0.0001 IRI-mice vs SHAM, *** *p* < 0.001 IRI +EVs mice vs SHAM and ### *p* < 0.001 IRI +EVs mice vs IRI-mice for CD45+ cell enumeration.

Immunofluorescence-staining of kidney cryo-sections showed that the IRI-mice had a significantly elevated deposition of collagen I and positivity for alpha-SMA, compared to the control (SHAM) mice. In the IRI-mice that were treated with HLSC-EVs, collagen I and alpha-SMA protein expression was significantly reduced (Figure 4). Moreover, treatment with EVs also reduced inflammatory cell infiltration, demonstrated by a significant reduction in the amount of CD45-positive cells (Figure 4), thus confirming the results of molecular analyses. 

As the development of fibrosis re-activates the genes that are involved in EMT [39], we evaluated the expression levels of genes that were involved in EMT process, such as *SNAI1* and *TWIST1*, in the renal tissue of IRI-mice. Moreover, we evaluated the gene expression levels of vimentin (*VIM*), a marker of mesenchymal cells, and of zonula occludens 1 (*ZO1*), an adhesion receptor that is expressed by the epithelial cells [6,40].

The expression levels of SNAI1, TWIST1, and VIM were reduced, while the epithelial marker ZO1 increased in IRI-mice that were injected with EVs (Figure 3C). These changes, with respect to the IRI-mice that were injected with the vehicle alone, were statistically significant for TWIST1, VIM, and ZO1 (Figure 3C). 

### 2.2. HLSC-EVs Effects on Renal Function, Morphology and Gene Expression Profile of IRI-Mice Sacrificed at Early Time Points

We demonstrated that IRI-mice that were treated with EVs immediately after injury showed an attenuation of CKD development at both the histological and molecular levels. To evaluate how EV-treatment may interfere with the development of fibrosis, we sacrificed IRI-mice that were injected with EVs or with the vehicle alone two or three days after the injury.

**Figure 5:**
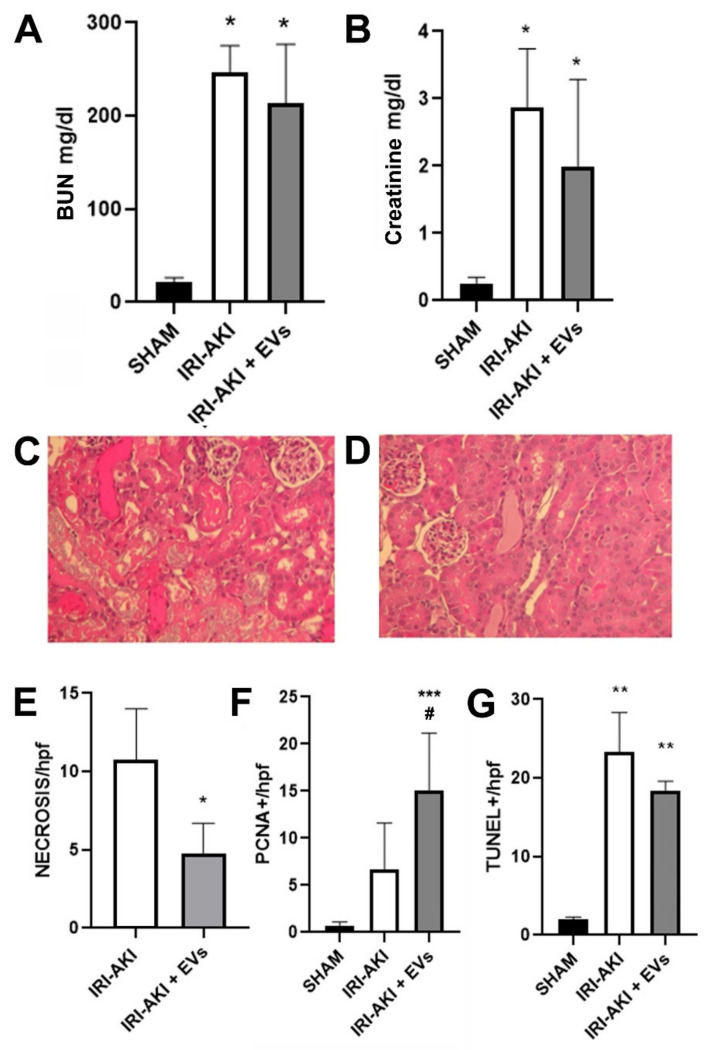
The effects of HLSC-EVs on kidney function and morphology in IRI-AKI mice that were sacrificed two days after the surgery. (**A**,**B**) BUN (**A**) and creatinine (**B**) plasma levels that are expressed as mg/dL were measured as biomarkers of renal function of SHAM mice (SHAM, *n* = 5) and of IRI-mice that were i.v.-injected with EVs (IRI-AKI + EVs, *n* = 5) or with the vehicle alone (IRI-AKI, *n* = 8) and sacrificed two days after the surgery. The data are represented as the mean ± SD and statistical analysis was performed using ANOVA with Tukey’s multicomparison test: * *p* < 0.05 IRI-AKI and IRI-AKI + EVs mice vs SHAM mice. (**C**,**D**) Representative light microscopy micrographs of renal histology of IRI-AKI mice (**C**) and or IRI-AKI + EVs mice (**D**) (original magnification: 400×). (**E**) Histological evaluation of tubular necrosis in 10 non-overlapping HPFs per section (original magnification: 400×) in IRI-AKI and IRI-AKI + EVs mice. The data are represented as the mean ± SD and statistical analysis was performed using a two tailed unpaired *t*-test: * *p* < 0.05 IRI-AKI + EVs vs IRI-AKI mice. (**F**,**G**) Histological evaluation of proliferating cell nuclear antigen (PCNA) (**F**) and terminal transferase-mediated dUTP nick-end labeling (TUNEL) (**G**) positive cells in 10 non-overlapping HPFs per section (original magnification: 400×) in SHAM, IRI-AKI and IRI-AKI + EVs mice. The data are represented as the mean ± SD and statistical analysis was performed using ANOVA with Tukey’s multicomparison test: *** *p* < 0.001 IRI-AKI + EVs vs SHAM mice and # *p* < 0.05 IRI-AKI vs IRI-AKI + EVs for PCNA; ** *p* < 0.001 IRI-AKI and IRI-AKI+EVs vs SHAM for TUNEL.

Mice that were sacrificed at day two post-surgery showed signs of AKI. In particular, an increase of BUN and creatinine plasma levels was detected (Figure 5A,B), together with the appearance of areas of tubular necrosis and luminal casts zones on histology (Figure 5C). EV administration induced a slight improvement of renal function and a significant amelioration of renal histology (Figure 5D), with a reduction in tubular necrosis (Figure 5E). Moreover, EV-treatment significantly increased the number of proliferating cells (Figure 5F) and slightly reduced the presence of apoptotic cells in the kidney (Figure 5G).

**Figure 6:**
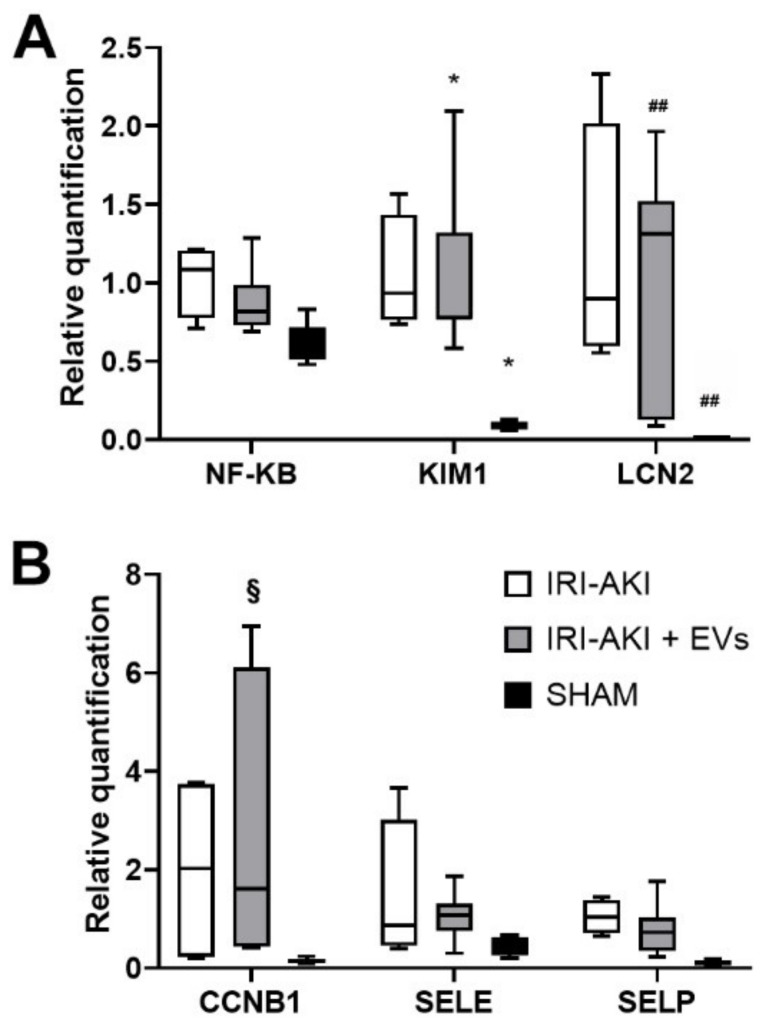
The effect of HLSC-EVs on renal damage in IRI-AKI mice that were sacrificed two days after the surgery. (**A**,**B**) Real-Time PCR analysis of the expression of genes that are related to inflammation (*NF-ΚB*) and to AKI (*KIM1* and *LCN2*) (**A**) and of genes that are related to proliferation (*CCNB1*) and to vascular cell adhesion (*SELE* and *SELP*) (**B**) in kidneys of SHAM mice (SHAM, *n* = 5), of IRI-AKI mice (IRI-AKI, *n* = 4), and of IRI-AKI mice that were treated with EVs (IRI-AKI + EVs, *n* = 5). All the mice were sacrificed 48 h after the surgery. The mean ± SD was calculated using ΔΔCt method, by comparing the gene expression levels of each group with the ones of the IRI-AKI group. Normalization was performed using *GAPDH* as the housekeeping gene. Statistical analysis was performed using ANOVA with Tukey’s multicomparison test: * *p* < 0.01 SHAM and IRI-AKI+EVs vs IRI-AKI mice for KIM-1; ## *p* < 0.001 SHAM and IRI-AKI+EVs vs IRI-AKI mice for LCN2 and § *p* < 0.002 IRI-AKI+EVs vs SHAM mice for CCNB1.

**Figure 7:**
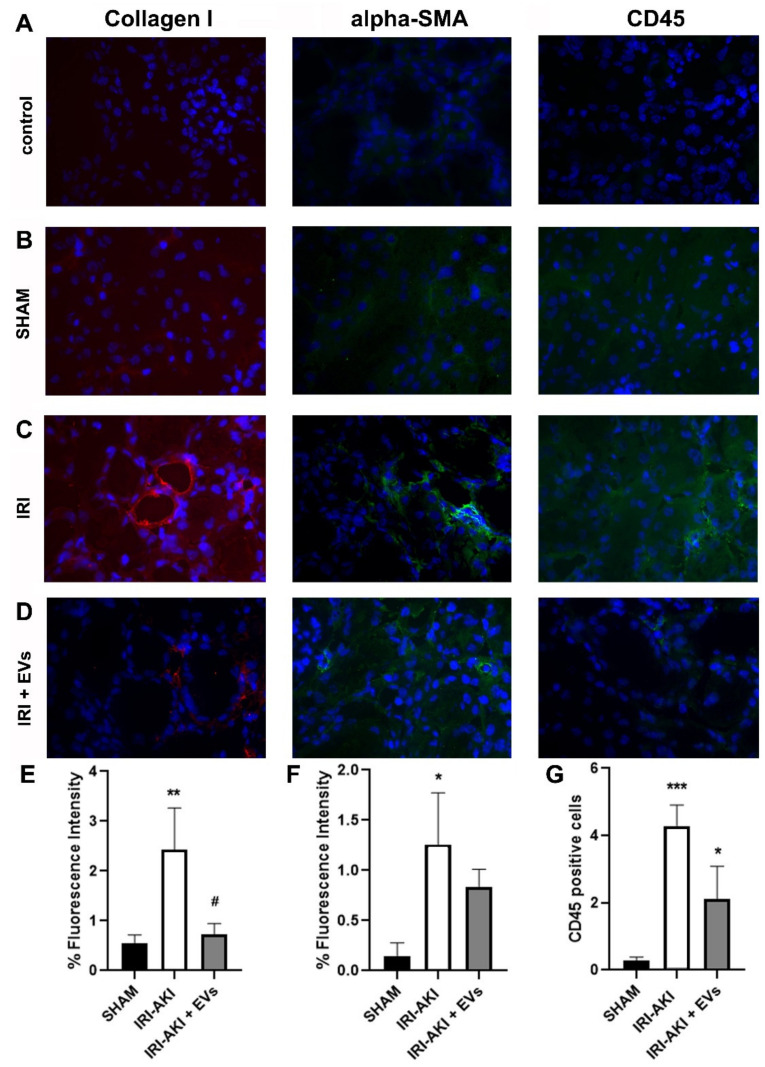
The effect of HLSC-EVs on fibrosis and inflammation in IRI-AKI mice that were sacrificed two days after the surgery. (**A**–**D**) Kidney cryo-sections from the control (SHAM) mice (**B**), IRI-mice that were treated with the vehicle (**C**), or with EVs (**D**) that were stained for collagen I, alpha-SMA, and CD45. Sections that were stained with secondary antibodies only served as control (**A**). (**E**–**G**) Histograms depicting the fluorescence intensity of collagen I (**E**) and alpha-SMA (**F**), and the quantification of CD45-positive cells (**G**) in renal cryo-sections from different experimental groups. The data represent the mean ± SD of the fluorescence intensity or cells that were positive per HPF measured from 10 images that were taken at random from four mice/groups (original magnification: 400×). Statistical analysis was performed using ANOVA with Tukey’s multicomparison test: ** *p* < 0.001 IRI-mice vs SHAM and # *p* < 0.02 IRI + EVs mice vs IRI-mice for % of fluorescence intensity for collagen I; * *p* < 0.01 IRI-mice vs SHAM for % of fluorescence intensity for alpha-SMA and *** *p* < 0.0008 IRI-mice vs SHAM and * *p* < 0.04 IRI + EVs vs IRI-mice and vs SHAM mice for CD45+ cell enumeration.

Real-Time PCR analyses showed a reduction in the expression levels of specific markers of AKI, such as kidney injury molecule-1 (*KIM1*) and lipocalin 2/NGAL (*LCN2*), and of endothelial damage, such as E-selectin (*SELE*) and P-selectin (*SELP*), in the EV-treated AKI mice (Figure 6A,B). In addition, EV-treatment slightly reduced the expression of the pro-inflammatory gene nuclear factor kappa-light-chain-enhancer of activated B-cells (*NF-KB*) (Figure 6A) and up-regulated the G2/mitotic-specific cyclin-B1 (*CCNB1*) (Figure 6B). Although the changes in the gene expression levels did not reach statistical significance after EV administration, an overall downward trend was observed in the expression of damage markers. In addition, the anti-inflammatory effect of EV-treatment was also indicated by the reduction of inflammatory cells that were accumulated in the kidney of the IRI-mice that were treated with EVs, as showed by immunofluorescence-staining with the leucocyte marker CD45 (Figure 7).

**Figure 8:**
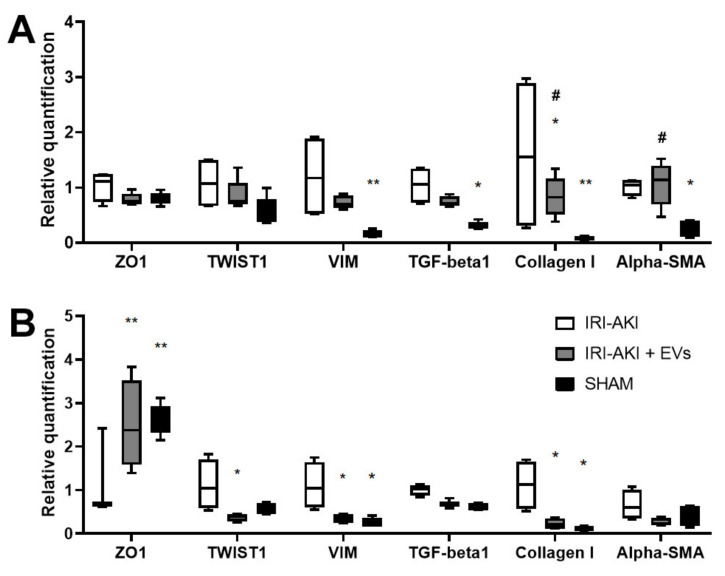
The effect of HLSC-EVs on renal fibrosis and EMT in IRI-AKI mice that were sacrificed two and three days after the surgery. (**A**,**B**) Real-Time PCR analysis of the expression of genes that are related to of EMT (*TWIST1, VIM*, and *ZO1*) and to fibrosis (*TGF-beta, collagen I*, and *alpha-SMA*) in kidney of SHAM (*n* = 5), of IRI-AKI (*n* = 4), and of IRI-AKI + EVs (*n* = 6) mice that were sacrificed two days after the surgery (**A**), and of SHAM (*n* = 5), of IRI-AKI (*n* = 4), and of IRI-AKI + EVs (*n* = 4) mice that were sacrificed three days after the surgery (**B**). The mean ± SD was calculated using ΔΔCt method, by comparing the gene expression levels of each group with the ones of the IRI-AKI group. Normalization was performed using *GAPDH* as the housekeeping gene. Statistical analysis was performed using ANOVA with Tukey’s multicomparison test: * *p* < 0.02, ** *p* < 0.001 SHAM and IRI-AKI + EVs vs IRI-AKI mice and # *p* < 0.01 IRI-AKI + EVs mice vs SHAM.

In IRI-mice that were injected with EVs and sacrificed two days after the injury, we also observed the reduction of the gene expression level of EMT markers (*TWIST1* and *VIM*) and of pro-fibrotic markers (*TGF-beta* and *collagen I*) (Figure 8A). In particular, the expression of *collagen I* in IRI-mice that were treated with EVs was significantly reduced compared to the IRI-mice that were injected with the vehicle alone. These reductions were also confirmed in the IRI-mice that were sacrificed at day three after the injury (Figure 8B). In this group of IRI-mice, the decrease of *TWIST1*, *VIM*, and *collagen I* was statistically significant with respect to the IRI-mice that were injected with the vehicle alone. At this time point, also the expression of alpha-SMA resulted down-regulated in the EV-treated mice. Besides, the expression of *ZO1* in IRI-mice that were treated with the vehicle alone was downregulated with respect to the SHAM mice, while in the IRI mice that were treated with EVs, it was maintained at levels that were similar to those of the SHAM mice. 

Immunofluorescence-staining of the kidney cryo-sections showed that the IRI-mice that were sacrificed at day two after the surgery had a significantly elevated deposition of type I collagen and of alfa-SMA positivity, compared to the control (SHAM) mice (Figure 7). In IRI-mice that were treated with HLSC-EVs collagen I and alfa-SMA were reduced, confirming the gene expression level detected by Real-Time PCR (Figure 7). 

## 3. Discussion

Although the kidney is able to recover from an ischemic or toxic injury, with the complete resolution of its normal structure and function, this is often not the case. Indeed, renal repair after damage can be maladaptive. AKI can lead to incomplete tubular repair, persistent tubulointerstitial inflammation, and a marked deposition of the extracellular matrix by myofibroblasts [1], which lead to long-term damage. For this reason, it is important to investigate those pathophysiological mechanisms that link AKI to the progression of CKD so that strategies can be developed that might counteract the development of fibrosis.

In this study, we set up a murine model of IRI that mimics both AKI and the development of CKD. We demonstrated that treatment with HLSC-EVs soon after injury interferes with the development of interstitial fibrosis and that the beneficial effect of EVs is already visible shortly after injury.

Previous research studies have reported that multiple weekly injections of HLSC-EVs significantly reduces the progression of fibrosis and the recruitment of inflammatory cells in murine models of CKD that are induced by aristolochic acid [32] and streptozotocin [28].

In IRI-mice that were sacrificed two months after injury, we showed that the administration of two doses of EVs (immediately after damage and three days later) can effectively reduce the development of interstitial fibrosis, both at histological and at the molecular levels, by down-regulating the expression of *alpha-SMA*, *collagen I*, and *TGF-beta* genes. Furthermore, the expression of pro-inflammatory genes *TNFα* and *IL-6* levels was significantly reduced by EV-treatment. These results indicate that an early EV administration to IRI-mice, soon after damage, is effective in attenuating the development of fibrosis and inflammation.

During kidney fibrosis, the presence of fibrogenic stimuli such as TGF-beta induces phenotypical changes in surviving TECs. These cells lose structural polarity and cell–cell-basement membrane contact, and acquire mesenchymal-like features, such as increased cell protrusions and motility [6]. This process, that is known as EMT, is characterized by the loss of epithelial adhesion markers, such as ZO1, which is found within adherens-type junctions and plays an important role in preserving the integrity and the polarization of the tubular epithelium [40]. Conversely, TECs up-regulate the expression of genes, such as *SNAI1* [41] and *TWIST1* [42], which are both expressed during early embryonic morphogenesis, and undergo a reorganization of the actin cytoskeleton. This involves the exchange of cytokeratin with VIM, the major intermediate filament of mesenchymal cells [40].

We examined the expression of EMT markers in kidney tissues two months after injury. In the IRI-mice, we found increased levels of the mesenchymal markers *SNAI1*, *TWIST1*, and *VIM* and a decreased expression of the epithelial marker *ZO1*. On the contrary, EV-administration restored the EMT genes in the IRI-mice to baseline levels: in fact, the levels of *TWIST1* and *VIM* were significantly downregulated, while the expression of *ZO1* was significantly increased. 

In IRI-mice that were sacrificed two days after injury, we found that HLSC-EVs induced a reduction in tubular necrosis, an increase in tubular cell proliferation, and a slight reduction in the expression levels of damage-related genes, in particular lipocalin-2/NGAL, considered a biomarker of renal proximal tubule injury [43]. EV-treatment slightly reduced the expression of endothelial damage-related markers (*SELE* and *SELP*) and of *NF-KB*, a transcription factor that controls the expression of pro-inflammatory factors and mediates cell proliferation and survival [44]. Furthermore, the up-regulation of *CCNB1* confirmed the pro-proliferative effect of HLSC-EVs that was observed at the histological level.

To investigate the capacity of EVs to interfere with the development of CKD, we also evaluated specific transcripts that were involved in fibrosis and EMT in mice that were sacrificed at early time points (two and three days after surgery and EV-treatment). The expression of the pro-fibrotic markers *alpha-SMA*, *collagen I*, and *TGF-beta* in AKI mice two days after IRI was higher than the expression of the same markers three days after IRI, compared to the SHAM controls. In IRI-mice that were sacrificed at day two, EV-treatment significantly reduced *collagen I* level. Following three days after IRI, mice that were treated with EVs showed an overall downward trend in the expression of fibrosis-associated markers.

The expression of EMT markers *VIM* and *TWIST1* in AKI mice that were sacrificed three days after IRI compared to the SHAM controls were more deregulated than the expression of the same markers two days after IRI. Interestingly, *VIM* is usually expressed in the metanephric mesenchyme during kidney development but not in the mature nephron and is re-expressed after IRI [45]. In IRI-mice that were sacrificed at early time points, *VIM* and *TWIST* gene expression was reduced by EV-treatment while *ZO1* expression increased. 

These data indicate that treatment with HLSC-EVs immediately after renal IRI protects the kidney from developing fibrosis by modulating the expression levels of specific genes that are involved in fibrosis and the EMT process. Moreover, it has recently been reported that HLSC-EVs protect the liver from IRI by modulating the gene expression of key inflammatory molecules [46]. Although these results hold great promise for a possible therapeutic approach against kidney injury, the complex mechanism by which HLSC-EVs can interfere with AKI and subsequently CKD development, remains to be elucidated. One limitation of this study is that the molecular content of HLSC-EVs has not yet been fully described. Recently, the characterization of the miRNA content of HLSC-EVs [28] has shown that these EVs contain several miRNAs targeting the expression of fibrosis- and EMT-related genes. Based on this assumption, we could then hypothesize that the effect of HLSC-EVs in the damaged kidney may be due to the transfer of specific anti-fibrotic non-coding RNAs into the renal cells, thus attenuating the ischemic damage and the subsequent progression to CKD.

## 4. Materials and Methods

### 4.1. HLSC Culture

HLSCs were isolated and maintained in a culture as previously described [34,47,48]. HLSCs were plated at a density of 2.5 × 10^5^ cells per flask (T75, Corning, VWR International, Milano, Italy) and maintained in the presence of Minimal Essential Medium (α-MEM, Lonza, Basel, Switzerland) with 10% Fetal Calf Serum (Gibco/Cambrex, Invitrogen, Carlsbad, CA, USA), specific growth factors (10 ng/mL of human recombinant Epidermal Growth Factor and of human recombinant Fibroblast Growth Factor basic, Miltenyi, Bergisch Gladbach, Germany), 2nM L-Glutamine (Lonza), and antibiotics (100U/mL of Penicillin/Streptomycin, Sigma-Aldrich, St. Louis, MO, USA).

### 4.2. Purification and Characterization of HLSC-EVs

The EVs were isolated from CM of sub-confluent HLSCs that were cultured in hyperflasks (Corning) at a density of 3000 cells/cm^2^ in α-MEM (Lonza) for 18 h. The next day, the cell supernatant was recovered, centrifuged at 3000× *g* for 15 min, and microfiltered with 0.22 µm filters to remove the cell debris. Then, the EVs were isolated by ultracentrifugation at 100,000× *g* for 2 h at 4 °C (Beckman Coulter Optima L-100 K, Fullerton, CA, USA). The EV-pellet that was obtained was resuspended in RPMI supplemented with 1% dimethyl sulfoxide (DMSO, Sigma-Aldrich) and stored at −80 °C until use in subsequent studies.

The concentration of the EV preparations was determined using the NanoSight LM-10 instrument (NanoSight Ltd., Amesbury, UK), equipped with a 405 nm laser. The recordings of three 60-s videos were examined by the Nanoparticle Tracking Analysis Software (NTA v. 3.4).

Transmission electron microscopy was performed to evaluate the size and integrity of HLSC-EVs, on approximately 3 × 10^9^ EVs that were placed on 200 mesh nickel formvar carbon-coated grids (Electron Microscopy Science, Hatfield, PA, USA). As previously described, the EVs were left to adhere for 20 min [49]. After EV adhesion, the grids were treated with 2.5% glutaraldehyde containing 2% sucrose and, after washes in distilled water, the EVs were negatively stained with NanoVan (Nanoprobes, Yaphank, NK, USA). The EV-preparations were observed using a Jeol JEM 1400 electron microscope (Jeol, Tokyo, Japan).

The EVs phenotype was characterized by flow cytometry analysis, using a bead-based multiplex analysis system (MACSPlex Exosome Kit, human, Miltenyi Biotec, Bergisch Gladbach, Germany), as previously reported [36,50]. Briefly, approximately 2 × 10^9^ EVs were diluted with MACSPlex buffer and incubated with MACSPlex Exosome Capture Beads for 18 h at 450 rpm, to allow EV binding to 39 different antibody-coated bead subsets. Counterstaining was carried out by incubating the EVs that were bound by capture beads with the APC-conjugated anti-CD63, anti-CD81, and anti-CD9 detection antibodies for 1 h at 450 rpm, in the dark. Washing steps with MACSPlex buffer were performed to remove the unbound antibodies. Approximately 5000 single beads per sample were acquired using a Cytoflex flow cytometer (Beckman Coulter, Brea, CA, USA). The CytExpert Software was employed to identify and gate all bead populations based on their respective fluorescence intensity. The median fluorescence intensity (MFI) was calculated for each capture bead subset and the background fluorescence intensity was removed by subtracting the MFI value of a blank control that was processed in the same way as the EV samples (medium + capture beads + detection antibodies) from the MFI of all the 39 capture bead subsets.

### 4.3. Western Blot Analysis

Cells and EV proteins were isolated by lysis for 30 min at 4 °C in RIPA buffer (20 nM Tris-HCl, 150 nM NaCl, 1% deoxycholate, 0.1% SDS, 1% Triton X-100, pH 7.8) in the presence of 1% phenylmethylsulfonyl fluoride, 1% protease and phosphatase inhibitor cocktail (Sigma-Aldrich). The BCA Protein Assay Kit (Pierce™ Thermo Fisher, Waltham, MA, USA) was used to quantify the protein concentration. The protein samples (10 µg) were loaded on 4–20% gradient Mini-PROTEAN TGX precast electrophoresis gels (Bio-Rad, Hercules, CA, USA), and separated under reducing conditions. The Trans-Blot Turbo Transfer System (Bio-Rad) was used to transfer the proteins onto 0.2-µm nitrocellulose membranes. The membranes were blocked in the presence of 0.1% Tween-20 and 5% bovine serum albumin for 2 h, then probed for 18 h at 4 °C with rabbit anti-GM130 (AbCam, Cambridge, UK), mouse anti-Alix, and mouse anti-CD63 (both from Santa Cruz Biotechnology, CA, USA). After extensive washing, the membranes were incubated for 1 h at room temperature with the appropriate peroxidase conjugated secondary antibody. Chemiluminescent signals were detected using the Chemidoc system after probing the membranes with the ECL substrate (Bio-Rad).

### 4.4. In Vivo Murine Model

Animal studies were performed in accordance with the National Institute of Health Guide for the Care and Use of Laboratory Animals. The Italian Health Ministry approved all procedures that were carried out on animals (authorization number: 870/2018-PR). 

Ten-week-old male BALB-c mice (Charles River Laboratories, Wilmington, MA, USA), weighing from 22 to 26 g, were anesthetized using an intramuscular (i.m.) injection of zolazepam 80 mg/kg and xilazina 16 mg/kg. To expose the left kidney, a small midline laparotomy was made under sterile conditions. A no traumatic vascular clamp was used to clench the renal pedicle for 30 min (Fine Science Tools, Foster City, CA, USA). A right nephrectomy was performed immediately after clamping. During the surgical procedures, the body temperature was maintained at 37 °C by placing the animals on a heating plate. After removing the clamp, reperfusion of the kidney was confirmed visually. The abdominal incision was closed with a 6-0 silk suture. All the animals were closely monitored after surgery, and ketorolac (5 mg/kg) was administered as an analgesic when required. 

HLSC-EVs (1 × 10^9^) were injected via IV -i.v. (tail vein) immediately after the surgery and after three days (Figure 2A). The mice were sacrificed two months after the surgery to evaluate CKD development, or two and three days after injury to evaluate AKI. 

### 4.5. Renal Function

To measure the plasma creatinine and BUN, blood samples were collected. The concentrations of creatinine were determined using a colorimetric microplate assay that was based on the Jaffe reaction (Quantichrome Creatinine Assay, BioAssay Systems, Hayward, CA, USA). The levels of BUN were evaluated by measuring serum urea using a colorimetric assay kit, following manufacturer’s protocol (Arbor Assays, Ann Arbor, MI, USA).

### 4.6. Histological Analyses

The kidney morphology was assessed using formalin-fixed paraffin-embedded tissue-staining. A total of five micrometer paraffin sections were routinely stained with hematoxylin and eosin (H&E, Merck) for microscopic evaluation, or Masson’s trichrome for collagen detection.

The surface area that was occupied by collagen fibers was quantified in 10 random non-overlapping HPFs per section from images that were taken at a magnification of 400×, and multiphase image analyses were carried out using ImageJ software.

The numbers of casts and necrotic tubules were quantified in a single-blind fashion in up to 10 nonoverlapping fields (for each section stained with H&E) using a 40× objective [12,13].

Apoptosis was evaluated using TUNEL assay (ApopTag Apoptosis Detection Kit; Millipore Inc., Billerica, MA, USA), as previously described [13]. The proliferation of the renal tubular cells was quantified by immunohistochemistry, as described previously [12,14]. TUNEL- and PCNA-positive cells were quantified by counting the number of positive nuclei per HPF (400×) in 10 randomly chosen sections of the kidneys.

Immunofluorescence was performed on cryostat sections. The sections were stained with mouse anti-collagen I (1:100, AbCam), mouse anti-alpha SMA (1:100, AbCam), and rabbit anti-CD45 (1:50, AbCam) for 1 h at room temperature. This was followed by Alexa Fluor 488, or Texas Red conjugated secondary antibody (Molecular Probes, Invitrogen)-staining for 1 h at room temperature. Sections that were stained with secondary antibodies only, served as a control. Hoechst 33258 dye (Sigma-Aldrich) was added for nuclear-staining. Fluorescent microscopy analysis was performed using a Zeiss Apotome Microscope (Carl Zeiss International, Jena, Germany). The surface area that was occupied by fluorescent signal was quantified in 10 random non-overlapping HPFs per section from the images that were taken at a magnification of 400×, and multiphase image analyses were carried out using ImageJ software. CD45+ cells were quantified by counting the number of positive cells per HPF (400×) in 10 randomly chosen sections of the kidneys.

### 4.7. Molecular Analyses

Total RNA was extracted from the renal tissue of the SHAM or IRI-mice that were treated or not with HLSC-EVs using TRIzol™ reagent (Ambion, Thermofisher, Waltham, MA, USA), following the manufacturer’s instructions. A Bullet Blender instrument (Next Advance Inc., New York, NY, USA) was used to homogenize TRIzol™ solutions using 0.5 mm zirconium oxide beads at a speed of 8 rpm for 3 min, followed by centrifuge at 12,000× *g* for 10 min at 4 °C. RNA that was purified from the supernatant of the homogenized tissue was quantified spectrophotometrically (mySPEC, VWR, Radnor, PA, USA).

A High Capacity cDNA Reverse Transcription Kit (Applied Biosystems, Foster City, CA, USA) was used to convert RNA into cDNA. A 96-well QuantStudio 12K Flex Real-Time PCR system (Thermo Fisher Scientific) was used to evaluate specific gene expression by quantitative Real-Time PCR (qRT-PCR). Each well was loaded with 20 µL reaction mixture containing Power SYBR Green PCR Master Mix (Applied Biosystems), 5 or 10 ng of sample cDNA, and sequence-specific oligonucleotide primers (100 nM, purchased from MWG-Biotech, Eurofins Scientific, Brussels, Belgium). All the primers that were used for qRT-PCR are listed in Table 1. To normalize the RNA inputs, *GAPDH* was used as housekeeping gene. For all samples, fold-change expression with respect to the IRI group or the SHAM group was calculated using ΔΔCt method.

### 4.8. Statistical Analyses

Data analyses were carried out using GraphPad Prism v.8.0. Results from the histological and molecular analyses are expressed as the mean ± SD. Statistical analyses were performed by employing Ordinary One way ANOVA with Tukey’s multiple comparison test and a *p*-value of < 0.05 was considered significant. For tubular necrosis statistical analyses was performed by employing a two tailed student *t*-test and a *p*-value of <0.05 was considered significant.

## Figures and Tables

**Table 1:** Primers used for qRT-PCR to evaluate gene expression.

Gene	Forward (5′ → 3′)	Reverse (3′ → 5′)
m-Alpha-SMA	CATCTCCGAAGTCCAGCACA	GACGCACCACTGAACCCTAA
m-CCNB1	GGATTCAAGTGCATTCTCTCAGTG	TCTGGCTGTCAGAATTCAAAGC
m-Collagen I	ACCTTGTTTGCCAGGTTCAC	ATCTCCCTGGTGCTGATGGAC
m-GAPDH	TGTCAAGCTCATTTCCTGGTA	TCTTACTCCTTGGAGGCCATGT
m-IL-1beta	CAACCAACAAGTGATATTCTCCATG	GATCCACACTCTCCAGCTGCA
m-IL-6	ACCAGAGGAAATTTTCAATAGGC	TGATGCACTTGCAGAAAACA
m-KIM1	ATGAATCAGATTCAAGTCTTC	TCTGGTTTGTGAGTCCATGTG
m-LCN2	TGCACAGGTATCCTCAGGTACAGA	GGAAAAATACCATGGCGAACTG
m-NF-KB	ACAGGTCAAAATTTGCAACTATGTG	TGCATACCCCGTCCTCACA
m-SELE	GTCTAGCGCTGGATGAAAG	ATCGCCACCAGATGTGTGTA
m-SELP	GGGCTCAACTCATCTGGTTC	CATTGAGGTGAGCGATTTCA
m-SNAI1	CTGCTTCGAGCCATAGAACTAAAG	GAGGGGAACTATTGCATAGTCTGT
m-TGF-beta	GCAACAATTCCTGGCGTTACC	CGAAAGCCCTGTATTCCGTCT
m-TNF-alpha	CATCTTCTCAAAATTCGAGTGACAA	TGGGAGTAGACAAGGTACAACCC
m-TWIST1	AGCGGGTCATGGCTAACG	GGACCTGGTACAGGAAGTCGA
m-VIM	GAAATTGCAGGAGGAGATGC	TCCACTTTCCGTTCAAGGTC
m-ZO1	GATCCCTGTAAGTCACCCAGA	CGCTCATCTCTTTGCACTACCA

## Abbreviations

IRI	ischemia and reperfusion injury
AKI	acute kidney injury
TECs	tubular epithelial cells
CKD	chronic kidney disease
EMT	epithelial-to-mesenchymal transition
TGF-beta	transforming growth factor-beta
MSC	mesenchymal stromal cell
EV	extracellular vesicle
MSC-EVs	EVs derived from MSCs
HLSC	human liver stem cell
CM	conditioned medium
HLSC-EVs	EVs derived from HLSCs
alpha-SMA	alpha-smooth muscle actin
BUN	blood urea nitrogen
ANOVA	analysis of variance
IL-6	interleukin-6
TNF-alpha	tumor necrosis factor-alpha
VIM	vimentin
ZO1	zonula occludens 1
PCNA	proliferating cell nuclear antigen
TUNEL	terminal transferase-mediated dUTP nick-end labeling
HPF	high-power field
KIM1	Kidney injury molecule 1
LCN2	lipocalin 2/NGAL
SELE	E-selectin
SELP	P-selectin
NF-KB	nuclear factor kappa-light-chain-enhancer of activated B-cells
CCNB1	G2/mitotic-specific cyclin-B1
MFI	median fluorescence intensity
qRT-PCR	quantitative Real-Time PCR
